# Resolution of anti-GAD-associated autoimmune encephalitis in patients treated with efgartigimod

**DOI:** 10.3389/fneur.2025.1550023

**Published:** 2025-04-28

**Authors:** Min Chen, Zhuajin Bi, Wenzhong Kang, Ruihan Liu, Liu Hongbo, Yan Jiang, Qun Wang

**Affiliations:** ^1^Department of Neurology, The First Affiliated Hospital of Zhengzhou University, Zhengzhou, China; ^2^Xiangya School of Medicine, Central South University, Changsha, China; ^3^Department of Neurology, Beijing Tiantan Hospital, Capital Medical University, Beijing, China; ^4^China National Clinical Research Center for Neurological Diseases, Beijing, China

**Keywords:** efgartigimod, autoimmune epilepsy, autoimmune encephalitis, GAD, treatment

## Abstract

**Introduction:**

Temporal lobe epilepsy (TLE) is a significant clinical phenotype of anti-glutamic acid decarboxylase (GAD)-associated disease, which is characterized by disturbances in GABAergic inhibitory neurotransmission. Efgartigimod, a neonatal crystallizable fragment receptor antagonist, controls the trafficking and recycling of pathogenic anti-GAD immunoglobulin G, showing promise as a therapeutic target.

**Methods:**

We present a case report involving the treatment of three patients affected by GAD-seropositive autoimmune encephalitis with efgartigimod. The patients’ overall disability was assessed using the modified Rankin scale.

**Results:**

After 4 weeks of efgartigimod treatment, the patients demonstrated substantial improvements, including no dementia or behavioral abnormalities and well-controlled seizures.

**Discussion:**

Our findings suggest that efgartigimod is a potential candidate drug for the treatment of anti-GADassociated autoimmune encephalitis, particularly in patients presenting with TLE.

## 1 Introduction

Glutamic acid decarboxylase (GAD) is an intracellular enzyme responsible for converting glutamate into gamma-aminobutyric acid (GABA), the brain’s primary inhibitory neurotransmitter. The presence of GAD antibodies reduces GABAergic transmission, resulting in a range of neurological symptoms, including stiff-person syndrome, autoimmune epilepsy, limbic encephalitis, cerebellar ataxia, progressive encephalomyelitis with rigidity and myoclonus, and eye movement disorders. Collectively, these conditions are known as GAD antibody-spectrum disorders (1).

Anti-GAD antibodies are frequently observed in patients with pharmaco-resistant epilepsy and convulsive or non-convulsive status epilepticus, particularly temporal lobe epilepsy (TLE) (2, 3). In a retrospective study, anti-GAD antibodies were detected in 22% of patients with various epilepsies attributed to autoimmune etiology (4). Conversely, among patients with temporal lobe epilepsy (TLE), the percentage of GAD-antibody positivity was reportedly even higher, up to 21.7% (5). Of interest, among 80 children with epilepsy, anti-GAD antibodies were the third most common antibody after anti-nuclear and anti-voltage gated potassium channel antibodies (5).

High titers of GAD antibodies in the serum and/or cerebrospinal fluid (CSF) can be used in diagnosis. Symptomatic or immunologic treatment strategies should be implemented to control seizures. High doses of glucocorticoid, intravenous Ig (IVIg), immune adsorption (IA), and plasma exchange (PE) are frequently used in the management of severe autoimmune diseases mediated by pathogenic immunoglobulin G (IgG) autoantibodies. Such IgG-modulating approaches are quite frequently associated with severe adverse reactions, even including lethal allergic shock, and place substantial burdens on patients due to their high cost and invasiveness. Neonatal Fc receptors (FcRns) recycle IgGs by preventing their lysosomal degradation. As this process also enhances the half-life of pathogenic auto-IgGs, several inhibitors of the IgG-FcRn interface have been conceived to treat autoimmune diseases (6). Recently, efgartigimod, a novel FcRn inhibitor, has emerged as a more effective, safer, and more convenient alternative for clearing pathogenic IgGs. By inhibiting the FcRn receptor, efgartigimod reduces the recycling of IgG antibodies, thereby lowering their concentration and mitigating the immune response (7). This approach offers a promising treatment option for patients with anti-GAD-associated autoimmune encephalitis (8). In this study, we describe the first data on the use of efgartigimod in patients affected with anti-GAD-seropositive encephalitis presenting with TLE.

## 2 Case reports

### 2.1 Case 1

**Figure 1:**
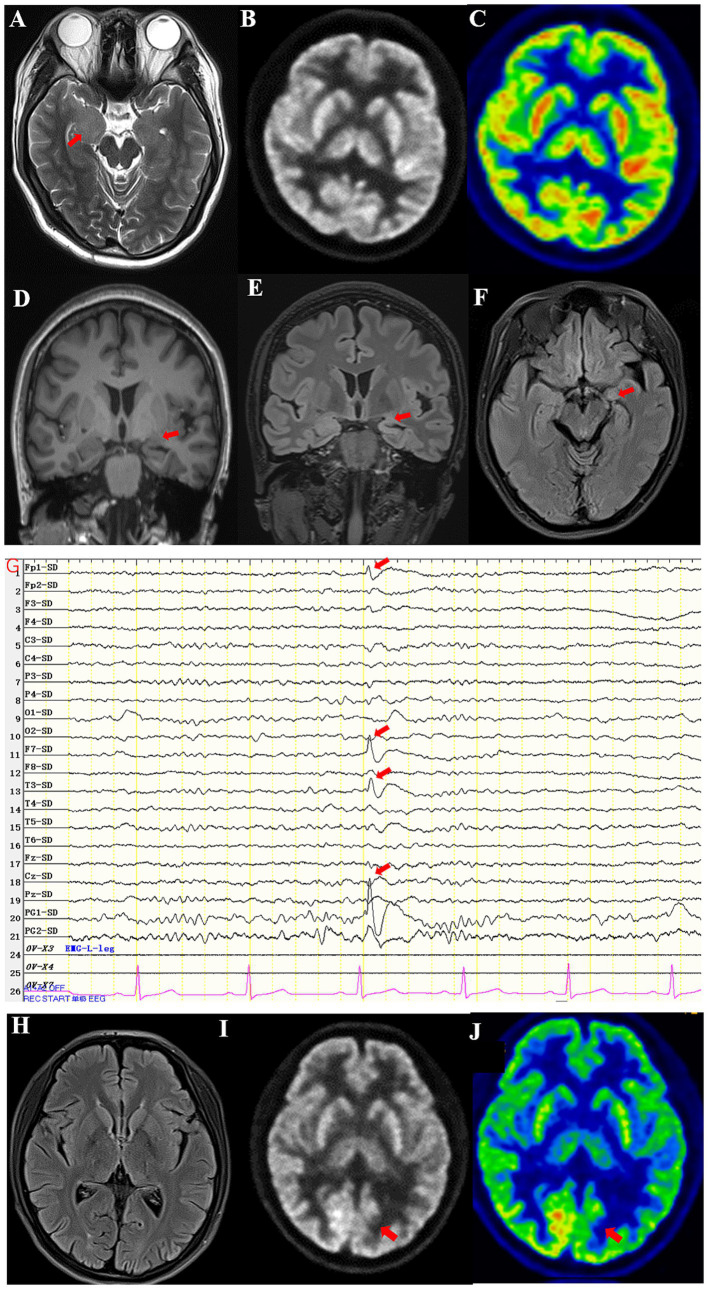
**(A)** High T2 signal in the right medial temporal lobe of Case 1. **(B,C)** PET-CT showed decreased FDG affinity in bilateral temporal lobes and cerebellar hemispheres of Case 1. **(D)** The volume of the left amygdala was smaller than that of the right amygdala in T1WI of Case 2. **(E)** Atrophy of the left amygdala in a coronal scan of Case 2. **(F)** Atrophy of the left amygdala in an axial scan of Case 2. **(G)** EEG showed episodic sharp wave complexes in the left PG1 of Case 2. **(H)** No abnormal signals were seen on the T2-FLAIR in coronal scan of Case 3. **(I,J)** PET-CT showed decreased FDG affinity in left inferior parietal lobe of Case 3.

A 22-year-old male patient with a normal medical history had cognitive impairment and three episodes of generalized tonic–clonic seizures in the past 3 months, each lasting for about 2 min. The seizures were associated with a loss of consciousness, foaming at the mouth, frothing, and tonic and clonic movements of all four limbs, without any prodromes, and were followed by a sustained reaction delay of about 5 min. The patient did not have typical absence seizures, but he had memory loss. Physical examination during the interval between seizures revealed the nervous system was normal. Mini-mental state examination (MMSE) and mini-mental cognitive assessment (MoCA) tests were performed on the day of hospitalization, and the scores were 27/30 and 23/30, respectively, with the deductions both in delayed recall. He scored 18 in the symbol digit modalities test (SDMT). No significant abnormalities were found in routine blood tests or liver and kidney function, blood electrolyte, blood ammonia, anti-nuclear antibody, and tumor marker screening. The patient underwent computer tomography (CT), and thymoma or other neoplasms were excluded. Magnetic resonance imaging (MRI) showed hypersignal in the right temporal lobe on T2WI (Figure 1A). Positron emission tomography-computed tomography (PET-CT) showed decreased FDG affinity in the bilateral temporal lobes and cerebellar hemispheres (Figures 1B,C). Electroencephalogram (EEG) examination showed no clear epileptic waves. CSF examination results indicated a slight increase in white blood cell count (12 × 10^6^/L), while the pressure, appearance, white blood cell count, sugar, chloride, and protein measurements of the CSF were normal. An oligoclonal IgG band was detected in the CSF but not the serum. The anti-GAD antibody titer in serum was 1:320 and that in CSF was 1:10. GAD antibodies were detected using indirect immunofluorescence and cell-based assays. Antibodies against the N-methyl-d-aspartate receptor (NMDAR), *γ*-amino butyric acid type B receptor (GABABR), *α*-amino-3-hydroxy-5-methyl-4-isoxazolepropionic acid receptor (AMPAR), and contact in associated protein 2 (CASPR2) were assayed, and the results were negative. Treatment with intravenous methylprednisolone (1,000 mg once a day) combined with levetiracetam tablets (twice a day, 1.5 g each time) and lacosamide tablets (twice a day, 100 mg each time) was administered 93 days after disease onset. Simultaneously, the methylprednisolone dosage was gradually reduced according to the Chinese expert consensus (9), i.e., 1,000 mg IV d1–d3, 500 mg IV d4–d6, 48 mg oral d7–d20, with dosage reduced by 4 mg every 2 weeks, but it failed to completely control the patient’s seizures. IVIg and PE were not administered because of the inaccessibility of the blood. IA was not available in our hospital due to the local medical insurance reimbursement policy in our province. Thus, starting from day 3, 10 mg/kg efgartigimod was administered intravenously once a week for 4 weeks. After 4 weeks of treatment, serum levels of anti-GAD antibody and IgG levels reduced from 1:320 to 1:20 and from 9.4 g/L to 3.8 g/L respectively, as described in Table 1. The patient’s epilepsy did not recur in the subsequent 3 months. Their EEG returned to normal. The patient showed improvements in MMSE and MoCA at the end of the third month, with scores of 30/30 for both tests. He scored 56 in the SDMT. No abnormal signs or hippocampal atrophy were observed in the brain MRI repeated at the end of the third month. His modified Rankin scale (mRS) score was 1.

**Table 1:** Serum tilter of anti-GAD antibody and serum levels of IgG in the three cases.

Cases	Before efgartigimod		After efgartigimod	
	Serum anti-GAD tilter (CBA)	Serum level of IgG (g/L)	Serum anti-GAD tilter (CBA)	Serum level of IgG (g/L)
Case 1	1:320	9.4	1:10	3.8
Case 2	1:100	8.1	1:32	3.2
Case 3	1:320	7.07	1:1	3.5

CBA, cell-based indirect immunofluorescent assay.

### 2.2 Case 2

A 43-year-old female patient with a normal medical history had dizziness and blurred vision, generalized tonic–clonic seizures and compound partial seizures with automatism over the past 5 years, each lasting for around 1 min. The seizures were associated with a loss of consciousness, foaming at the mouth, frothing, and tonic and clonic movements of all four limbs, without any prodromes, and were followed by a sustained reaction delay of about 3 min. She did not have memory loss. On physical examination, the nervous system during the interval between seizures was normal. MMSE and MoCA tests were performed on the day of hospitalization, and the scores were 30/30 and 30/30, respectively. No significant abnormalities were found in routine blood tests or liver and kidney function, blood electrolyte, blood ammonia, anti-nuclear antibody, and tumor marker screening. MRI showed atrophy in the left hippocampus and amygdala (Figures 1D–F). EEG examination showed episodic sharp wave complexes in the left PG1 (Figure 1G). The CSF examination results indicated the white blood cell count (2 × 10^6^/L), pressure, appearance, sugar, chloride, and protein were normal. An oligoclonal IgG band was detected in the CSF but not the serum. The anti-GAD65 antibody titer in serum was 1:100 and that in CSF was 1:10. GAD was detected using indirect immunofluorescence and cell-based assays. Assays for antibodies against NMDAR, GABABR, AMPAR, and CASPR2 were negative. Five years after disease onset, intravenous methylprednisolone (IVMP) was administered at a dose of 1,000 mg per day for pulse therapy over 3 days, followed by a reduced dose of 500 mg per day for an additional 3 days. Then, IVMP was switched to daily prednisone, starting at 50 mg and tapering by 5 mg every 2 weeks. The patient’s seizures were not controlled well though, and a combination of oxcarbazepine tablets (twice a day, 0.45 g each time) and perampanel (4 mg once a day) was used. Thus, starting from day 21, 10 mg/kg efgartigimod was administered intravenously once a week for 4 weeks. After 4 weeks of treatment, serum levels of anti-GAD antibody and IgG decreased, as shown in Table 2. The patient’s epilepsy did not recur in the next 3 months. Her mRS score was 1.

**Table 2:** Clinical characteristics of the patients.

No.	Sex	Age, y	Comorbidities	Clinical features	MRI findings	Treatments	Serum antibody titer		Outcome
							Admission	After 4 weeks of efgartigimod treatment	
Case 1	Male	22	None	Temporal lobe epilepsy	T2 hypersignal in right temporal lobe	IVMP, Efgartigimod, Levetiracetam, and Lacosamide	1:320	1:10	Good
Case 2	Female	43	None	Temporal lobe epilepsy	T2 hypersignal in bilateral hippocampal; Atrophy of the left hippocampal and amygdala	IVMP, Efgartigimod, Oxcarbazepine, and Perampanel	1:1000	1:32	Good
Case 3	Female	20	DM1	Epilepsy	Normal	Efgartigimod, valproic acid, lamotrigine, levetiracetam, lacoxamine	1:100	1:1	Improved

DM1, diabetes mellitus type 1; IVMP, intravenous methylprednisolone; MRI, magnetic resonance imaging.

### 2.3 Case 3

A 20-year-old female patient with type 1 diabetes had paroxysmal absence seizures for 7 years. During the attacks, she lost consciousness, smacked her lips, and chewed for 30 s. Valproic acid (500 mg, twice a day), lamotrigine (100 mg, twice a day), levetiracetam (1,500 mg, twice a day) and lacosamide (100 mg, twice a day) were used and reached effective blood concentration, but seizures occur 2–3 times a month. Sometimes she had hypoglycemia, with the lowest level being 2.9 mmol/L. Her blood sugar was 3.8 mmol/L, and glycosylated hemoglobin was 5.6%. The antibody titer for GAD was 1:320 in serum and 1:10 in CSF, and the antibody for islet cells was elevated to 48 COI (normal range 0–1). There was no abnormal signals in the MRI (Figure 1H). PET-CT showed lower affinity in the bilateral lateral frontal lobe, left medial frontal lobe, bilateral posterior cingulate gyrus, bilateral anterior cuneiform lobe, and left inferior parietal lobe (Figures 1I,J). EEG examination showed no clear epileptic waves. The MRI of her upper abdomen showed atrophy of the pancreas. She was treated with efgartigimod once a week for 4 weeks. Her seizures were reduced to once every 2 months, and her blood sugar was stable, with no episodes of hypoglycemia. Her mRS score was 1.

Dynamic changes of GAD antibody titer and IgG levels before and after the treatment were shown in Table 2.

## 3 Discussion

GAD is an enzyme critical for converting glutamate into GABA, a major inhibitory neurotransmitter in the brain. GAD-antibody-associated autoimmune encephalitis refers to a rare neurological condition in which GAD antibodies attack parts of the brain, particularly regions involved in memory, movement, and cognition, leading to various neurological disorders such as memory loss, seizures, mood changes, cognitive impairment movement disorders, and sleep disturbances. It has been proposed that a cellular neurotoxic effect against GABAergic interneurons plays a role in the mechanism of GAD-epilepsy due to the finding of cytotoxic T lymphocytes in temporomesial tissue biopsies from some GAD-positive patients with pharmaco-resistant epilepsy (9, 10).

Seizures are common in anti-GAD encephalitis due to decreased GABAergic function, and they are often refractory. The early administration of immunotherapy is crucial to prevent permanent brain tissue damage and effectively control epilepsy. Treatments for TLE include anti-seizure medicines combined with corticosteroids, IVIg, PE, and IA. Corticosteroids are the preferred treatment for autoimmune encephalitis due to their strong anti-inflammatory and immune regulatory abilities, as well as their convenience and economic advantages. However, their long-term and extensive use has considerable side effects, such as femoral head necrosis and obesity. Moreover, they require a gradual reduction for discontinuation. IVIg, a preparation of highly purified polyclonal immunoglobulins derived from an average of 1,000–15,000 healthy donor serum samples per batch (11), is known to exert immunomodulatory effects through various pathways, including neutralizing antibodies, Fc receptor modulation, anti-inflammatory effects, and autoantibody regulation (11, 12). However, the potential risks, including kidney damage, allergic reactions, and thromboembolism, and its inherent scarcity limit its use (11, 12). Reports indicate that combination therapies lead to more favorable outcomes for epilepsy and better neurological deficits scores than corticosteroid alone in autoimmune-associated epilepsy (13). Efgartigimod was formulated for the treatment of autoimmune diseases and has been approved for the treatment of myasthenia gravis, in which pathogenic IgG plays an important mechanistic role. Its mechanism of action involves targeting and reducing the levels of IgG antibodies to regulate the overactivity of the immune system. Specifically, efgartigimod is an FcRn inhibitor, and by inhibiting the FcRn receptor, it reduces the recycling of IgG, thus lowering the concentration of IgG antibodies in the body. This drug is particularly useful in treating certain autoimmune diseases, as many of these diseases are associated with the hyperactivity of pathogenic IgG antibodies. Efgartigimod has already been used to treat other autoimmune disorders apart from myasthenia gravis, such as neuromyelitis optic spectrum disorder (14) and anti-LGI1-associated autoimmune encephalitis (15). Evidence indicates that efgartigimod can reduce relapses (16, 17), improve outcomes by lowering the levels of pathogenic IgG antibodies in IgG-mediated autoimmune disorders, minimize side effects, and decrease glucocorticoid levels (8).

Herein, we reported three cases that presented with TLE caused by GAD-antibody-associated autoimmune encephalitis. They were similar in clinical presentation, with all involving TLE and high titers of GAD in the sera. The first patient had cognition impairment and TLE, the second patient had TLE, while the third patient had TLE accompanied with type 1 diabetes. Methylprednisolone combined with anti-seizure medicines were used but failed to control TLE in cases 1 and 2. Patient 3 refused to use methylprednisolone because of her diabetes. Based on the theoretical framework and clinical practice, we decided to add efgartigimod to the treatment regimen when our patients continued to experience seizures despite corticosteroid use. To our knowledge, the present case report describes the first attempts to use efgartigimod to treat anti-GAD-associated autoimmune encephalitis presenting with TLE. In all three cases, efgartigimod combined with or without methylprednisolone resulted in favorable outcomes.

The use of IVMP was relatively late in Case 1. It is generally accepted that immunotherapy initiated beyond 4 weeks post-onset correlates with a poorer prognosis. However, this study highlights the positive effects of efgartigimod as a treatment for GAD65-positive autoimmune encephalitis. Despite the delay in the initiation of IVMP, the patient responded well to efgartigimod, which demonstrates that it may still be effective in cases where first-line immunotherapy, such as IVMP, has been initiated late.

## 4 Conclusion

To our knowledge, the present case report describes the first attempted use of efgartigimod to treat anti-GAD-associated autoimmune encephalitis presenting with TLE. The combination of methylprednisolone and efgartigimod eventually yielded good outcomes. While our findings are promising, they are based on a limited number of cases. Future research, particularly multicenter randomized controlled trials, is needed to further explore and validate the efficacy of efgartigimod in treating anti-GAD-associated autoimmune encephalitis.

## Data Availability

The original contributions presented in the study are included in the article/supplementary material, further inquiries can be directed to the corresponding authors.
